# VRK1 promotes cisplatin resistance by up-regulating c-MYC via c-Jun activation and serves as a therapeutic target in esophageal squamous cell carcinoma

**DOI:** 10.18632/oncotarget.20020

**Published:** 2017-08-07

**Authors:** Zhen-Chuan Liu, Kuo Cao, Zhao-Hua Xiao, Liang Qiao, Xue-Qing Wang, Bin Shang, Yang Jia, Zhou Wang

**Affiliations:** ^1^ Department of Thoracic Surgery, Shandong Provincial Hospital Affiliated to Shandong University, Jinan 250021, China

**Keywords:** esophageal squamous cell carcinoma, vaccinia-related kinase 1, cisplatin, c-Jun, c-MYC

## Abstract

Esophageal squamous cell carcinoma (ESCC) is a common malignant disease characterized by poor prognosis. Chemoresistance remains a major cause of ESCC relapse. Vaccinia-related kinase 1 (VRK1) has previously been identified as a cancer-related gene. However, there is little research demonstrating an association between VRK1 and ESCC. In this study, we show that VRK1 is overexpressed in ESCC primary tumor samples and cell lines. VRK1 expression was significantly correlated with clinical characteristics and predicted poor outcomes in ESCC patients. Functionally, knockdown of VRK1 inhibited ESCC cell proliferation, survival, migration and invasion; conversely, VRK1 overexpression produced the opposite effects. Furthermore, we found that up-regulation of VRK1 promoted cisplatin (CDDP) resistance in ESCC both *in vitro* and *in vivo*, whereas knockdown of VRK1 reduced this resistance. Further studies verified that VRK1 phosphorylated c-Jun and that the VRK1/c-Jun pathway contributed to CDDP resistance in ESCC. Mechanistically, a dual luciferase reporter assay revealed that c-Jun transcriptionally activated the expression of c-MYC. Silencing c-MYC abolished the c-Jun-mediated CDDP resistance of ESCC cells. A Kaplan-Meier analysis indicated that c-MYC is a potential prognostic factor in ESCC. Finally, luteolin, a VRK1 inhibitor, attenuated the malignant biological behaviors and CDDP resistance in ESCC cells. Collectively, we conclude that VRK1 promotes CDDP resistance through c-MYC by activating c-Jun and potentiating a malignant phenotype in ESCC. Our studies provide novel insight into the role of VRK1 in carcinogenesis and indicate that VRK1 can serve as a potential therapeutic target in ESCC.

## INTRODUCTION

Esophageal carcinoma is the eighth most common malignant disease and ranks as the sixth leading cause of cancer-related death worldwide [1]. Esophageal squamous cell carcinoma (ESCC), the predominant histological subtype of esophageal carcinoma, accounts for approximately 90% of newly diagnosed esophageal cancers in developing country and is most prevalent in the “esophageal cancer belt” stretching from Iran through central Asia to northeast China [2, 3]. Although enormous progress has been made in the diagnosis and treatment of ESCC, the 5-year survival rate for ESCC patients is only 5%–40% because of the propensity of ESCC to extensively spread and its low sensitivity to chemotherapy or targeted therapies [4, 5].

VRK1 (vaccinia-related kinase 1) is one of the three members of the VRK family of Ser-Thr kinases that participate in cell division, transcriptional activation, DNA repair and histone modification [6–9]. As a kinase, VRK1 can stabilize and activate numerous transcription factors, such as ATF2, CREB, p53 and histone H3, via phosphorylation [10–13]. In addition, VRK1 promotes the formation of 53BP1 foci in response to ionizing radiation-induced DNA damage and participates in the DNA damage response (DDR) by phosphorylating H2AX and NBS1 [7, 14, 15]. Up-regulation of VRK1 has been observed in various human cancers, and a high level of VRK1 at the protein or RNA level is associated with a proliferative phenotype. In p53-mutant lung carcinomas, high VRK1 levels due to loss of the p53-VRK1 auto-regulatory loop can contribute to tumor proliferation and progression [16]. In breast cancer, VRK1 depletion can inhibit cancer cell proliferation *in vitro* as well as growth and metastasis *in vivo* [17]. However, the exact clinical and biological role of VRK1 in the initiation and progression of ESCC remains unknown.

The oncogene c-Jun was initially identified as Fos-associated protein p39, which acted as a transcription factor by forming dimers with Jun-related protein, Fos-related protein or ATF/CREB protein family members to activate AP-1 [18, 19]. In cells, c-Jun phosphorylation is rapidly and transiently induced by numerous extracellular stress stimuli, which are mediated and relayed by mitogen-activated protein kinases (MAPKs), such as ERK1/2, p38K and c-Jun N-terminal kinase (JNK) [20–22]. Phosphorylated c-Jun becomes transcriptionally active and participates in cell proliferation, differentiation and apoptosis by controlling the expression of relevant genes [23]. A previous study discovered that VRK1 can phosphorylate c-Jun at serine 63 and serine 73 and interact with JNK [24]. Thus, aberrant VRK1 expression might induce constitutive c-Jun activity independent of extracellular stress or upstream signals and eventually contribute to oncogenesis.

Although previous research has reported that VRK1 expression levels are elevated in ESCC [25], the biological functions of VRK1 in ESCC are still unclear. In this study, we investigate the expression profile of VRK1 in ESCC and its correlation with clinicopathological characteristics. VRK1 was found to promote cell proliferation and migration as well as cisplatin (CDDP) resistance in ESCC. Furthermore, our results indicated that VRK1 up-regulates c-MYC through c-Jun activation and that this axis is responsible for VRK1-mediated CDDP resistance. We also examined the inhibitory effect of VRK1 using luteolin, a VRK1 inhibitor, in ESCC cells both *in vitro* and *in vivo*. These data indicate that VRK1 may serve as a therapeutic target for ESCC treatment.

## RESULTS

### VRK1 is up-regulated and associated with poor prognosis in ESCC

**Figure 1:**
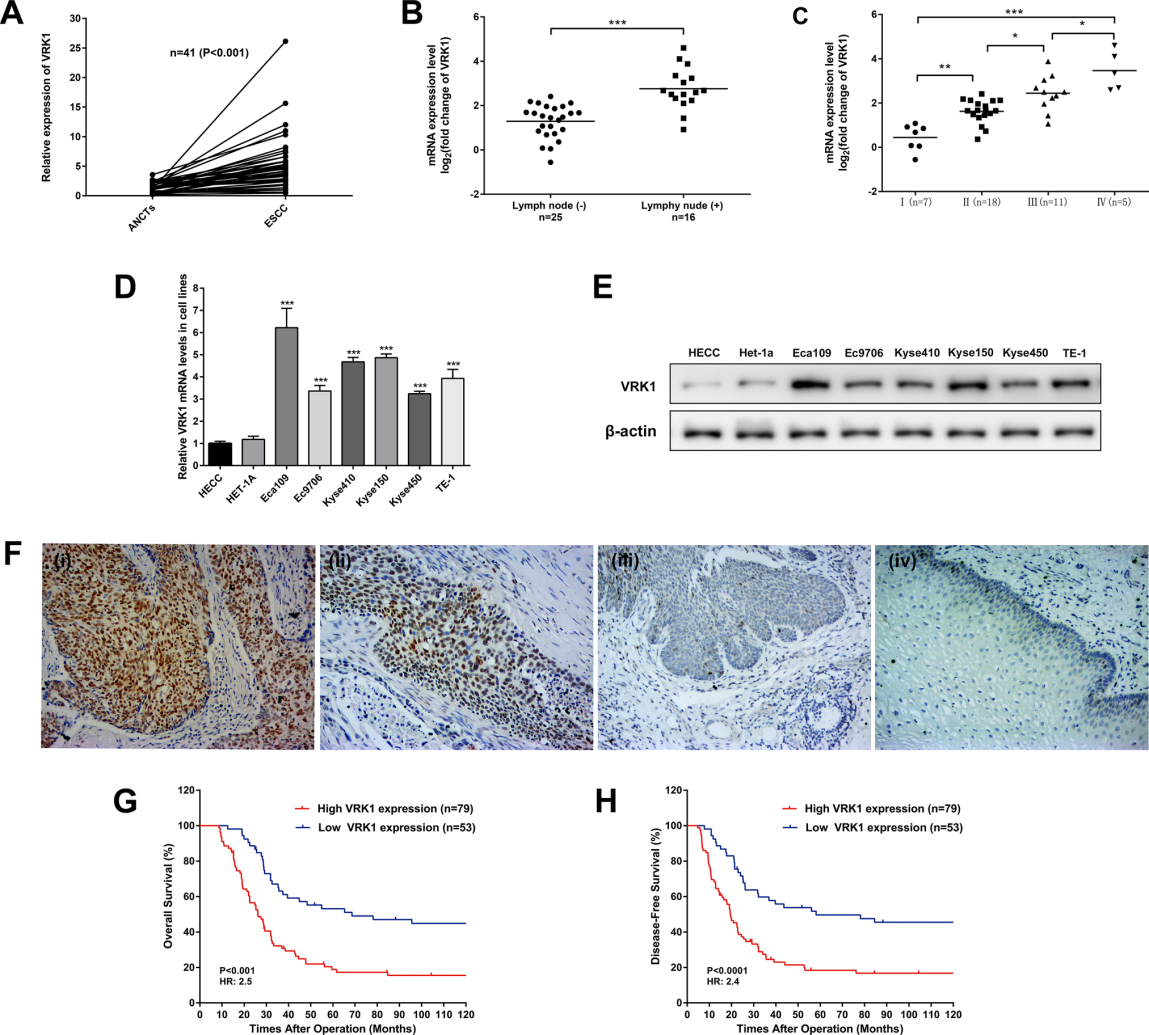
VRK1 is overexpressed in ESCC and correlated with the clinical outcome of ESCC patients (**A**) VRK1 expression levels in 41 paired ESCC and adjacent non-cancerous tissues (ANCTs) were analyzed via pair-wise Student’s *t*-test (*P* < 0.001). β-Actin was used as an internal control. (**B**) VRK1 mRNA levels in patients with lymphonodus involvement were higher than in patients without lymphonodus involvement in 41 cases (*P* < 0.001, Student’s *t*-test). (**C**) VRK1 mRNA levels were significantly associated with TNM stage in 41 of the ESCC patients (*P* < 0.001, one-way ANOVA). (**D, E**) The mRNA (D) and protein (E) levels of VRK1 in six ESCC cell lines (Eca109, Ec9706, TE-1, Kyse410, Kyse150 and Kyse450) and two normal esophageal epithelial cell lines (Het-1a and HECC). (**F**) Representative IHC staining of ESCC tissues with high VRK1 expression (i), moderate VRK1 expression (ii) and low VRK1 expression (iii). Weak staining in basal cells was seen in normal esophageal tissues (iv). Magnification: ×200. (**G, H**) A total of 132 ESCC cases were classified into high (SI ≥ 8) and low (SI < 8) VRK1 expression groups according to the staining index (SI). Kaplan-Meier survival analysis showed that high expression correlated with short OS (G) and DFS (H). The results are expressed as the mean ± SD of three independent experiments; *P < 0.05, **P < 0.01, and ***P < 0.001.

We examined gene profiling data for VRK1 expression in 41 paired fresh ESCC tissues using RT-PCR. Compared with the adjacent non-cancerous tissues (ANCTs), the tumor tissues presented significant VRK1 overexpression (Figure 1A). The VRK1 levels were associated with lymphatic metastasis (*P* < 0.001) and TNM stage (*P* < 0.001) (Figure 1B, 1C). Subsequently, we examined the VRK1 mRNA and protein levels in normal esophageal epithelial cells and in a panel of ESCC cell lines and found that VRK1 was differentially up-regulated in the ESCC cell lines compared with that in the normal Het-1a and HECC cells (Figure 1D, 1E).

The VRK1 protein level was further examined in 132 paraffin-embedded, archived ESCC tissues using immunohistochemistry (IHC). Positive staining was reflected by yellow or brown particles, which were predominantly aggregated in the nucleus. The semi-quantitative IHC analysis score for staining intensity and the percentage of positively stained cells showed that high VRK1 expression (staining index (SI) ≥ 8) was observed in 59.84% (79/132) of tumor tissues (Figure 1F). Consistent with this observation, statistical analysis revealed that aberrant VRK1 protein levels were significantly correlated with the depth of invasion, lymphatic involvement and TNM stage (Table 1). Furthermore, Kaplan-Meier and log-rank analyses indicated that patients with high levels of VRK1 expression (SI ≥ 8) had worse overall survival (OS) or disease-free survival (DFS) than patients with low levels of VRK1 (SI < 8) expression (Figure 1G, 1H). Ultimately, the univariate and multivariate Cox regression analyses demonstrated that VRK1 expression, along with T classification and N classification, were independent prognostic factors in ESCC (Table 2).

**Table 1:** Association between VRK1 protein level and several clinical characteristics in 132 ESCC cases

Demographic and clinical parameters	VRK1 Expression		*P-*value
	Low	High	
Sex			
Male	36	59	0.3967
Female	17	20	
Age			
≤ 60	30	52	0.2845
> 60	23	27	
Differentiation			
G1	21	17	0.0731
G2	24	44	
G3	8	18	
T stage			
T1	8	3	0.0081
T2	16	14	
T3/T4	29	62	
N stage			
N0	39	43	0.0204
N1	11	18	
N2/3	3	18	
TNM Stage			
I	18	10	0.0070
II	24	39	
III/IV	11	30	

Statistical analysis was performed using a Fisher’s exact test. *P*-values < 0.05 were considered significant.

**Table 2:** Univariate and multivariate Cox regression analysis for overall survival and disease-free survival in 132 ESCC patients

Variables	Overall survival					Disease-free survival						
	Univariate analysis		Multivariate analysis				Univariate analysis		Multivariate analysis			
	HR(95%CI)	*P*-value		HR(95%CI)	*P*-value	HR(95%CI)		*P*-value		HR(95%CI)	*P*-value	
T classification												
T1-T2;T3-T4	2.345(1.419–3.877)	**0.001**		1.954(1.100–3.469)	**0.022**	2.279(1.379–3.767)		**0.001**	1.841(1.043–3.248)		**0.035**	
N classification												
N0;N1-N3	2.202(1.451–3.342)	**< 0.001**		2.376(1.147–4.922)	**0.020**	2.206(1.454–3.348)		**< 0.001**	2.139(1.039–4.402)		**0.039**	
TNM stage												
I–II;III–IV	2.061(1.340–3.170)	**0.001**		0.766(0.346–1.695)	**0.511**	2.143(1.393–3.297)		**0.001**	0.893(0.408–1.955)		0.778	
VRK1 level												
High;low	2.691(1.706–4.245)	**< 0.001**		2.204(1.385–3.506)	**0.001**	2.614(1.657–4.124)		**< 0.001**	2.159(1.357–3.433)		**0.001**	

Statistical analysis was performed using a proportional hazard model (COX). *P*-values < 0.05 were considered significant. CI confidence interval, HR hazard ratio.

### VRK1 mediates invasion and proliferation of ESCC cell lines

**Figure 2:**
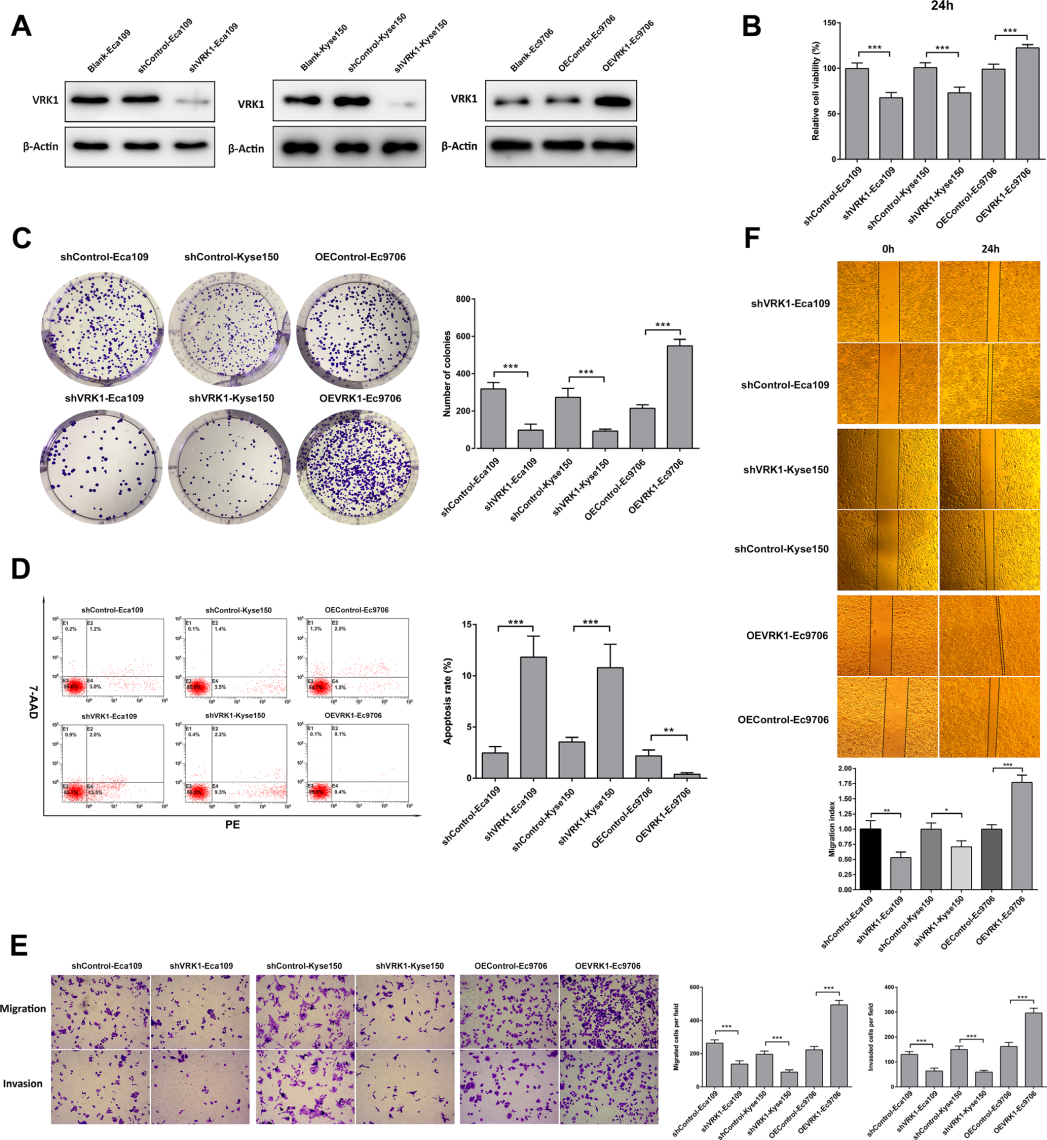
VRK1 is essential for the proliferation, survival, migration and invasion of ESCC cells (**A**) Western blot showing VRK1 expression in Ec109 (left) and Kyse150 (middle) cells transfected with VRK1-shRNA lentivirus or Ec9706 cells constitutively expressing full-length VRK1 cDNA. β-Actin served as an internal control. (**B**) CCK-8 assay showing that knockdown of VRK1 suppressed, whereas overexpression of VRK1 promoted, the proliferation and viability of ESCC cells. (**C**) Representative images (left) and quantification analysis (right) of colony formation of the indicated ESCC cells. (**D**) Apoptosis analysis of the indicated ESCC cells using flow cytometry. (**E**) Representative images and quantification analysis of migratory or invasive behavior of the indicated ESCC cells. (**F**) Representative images (upper) and quantification analysis (lower) of a wound-healing assay of the indicated ESCC cells. The migration index represents migration speed in relative to control group. Statistical analyses were performed using Student’s *t*-test. The results are expressed as the mean ± SD of three independent experiments; *P < 0.05, **P < 0.01, and ***P < 0.001.

Given that VRK1 expression was correlated with malignant clinical parameters (depth of invasion, lymphatic involvement and TNM stage) in ESCC patients, we therefore investigated the biological role of VRK1 in initiation and progression of ESCC. To explore the effect of VRK1 on the malignant ESCC phenotype, Ec9706 cells stably overexpressing full-length VRK1 DNA (OEVRK1-Ec9706) as well as Eca109 (shVRK1-Eca109) and Kyse150 (shVRK1 -Kyse150) cells transfected with VRK1-shRNA lentivirus were constructed. The efficiency of VRK1 knockdown or overexpression in ESCC cells was determined by western blotting (Figure 2A).

As expected, the proliferative capacity and colony-forming ability of Kyse150 and Eca109 cells transfected with shRNA targeting VRK1 were impaired compared with that of control cells, as determined with CCK-8 (Figure 2B and Supplementary Figure 1) and colony formation (Figure 2C) assays, respectively. In contrast, ectopic expression of VRK1 in Ec9706 cells led to a significant increase in cell proliferation (Figure 2B, Supplementary Figure 1) and colony formation (Figure 2C). Additionally, an apoptosis assay (Figure 2D) revealed significantly decreased apoptosis in VRK1-overexpressing cells (OEVRK1-Ec9706) and increased apoptosis in VRK1-knockdown cells (shVRK1 -Eca109 and shVRK1 -Eca109) compared with that in control cells.

We next examined the effects of VRK1 on ESCC cell migration and invasion. A Transwell assay showed that VRK1 overexpression and knockdown significantly enhanced and suppressed, respectively, the migratory and invasive ability of ESCC cells (Figure 2E). Similar results were obtained from a wound-healing assay (Figure 2F). These data demonstrated that VRK1 promoted malignant biological behavior in ESCC cells.

### VRK1 induces CDDP resistance in ESCC *in vitro* and *in vivo*

Cisplatin (CDDP) either alone or in combination with other cytotoxic drugs such as 5-flurouracil is used clinically in neoadjuvant or adjuvant chemotherapy for ESCC [26]. However, the poor response of these patients to CDDP remains a challenging clinical problem. Because VRK1 can enhance the proliferative and anti-apoptotic abilities of ESCC, we assessed the association between VRK1 expression and CDDP resistance in ESCC cells.

**Figure 3:**
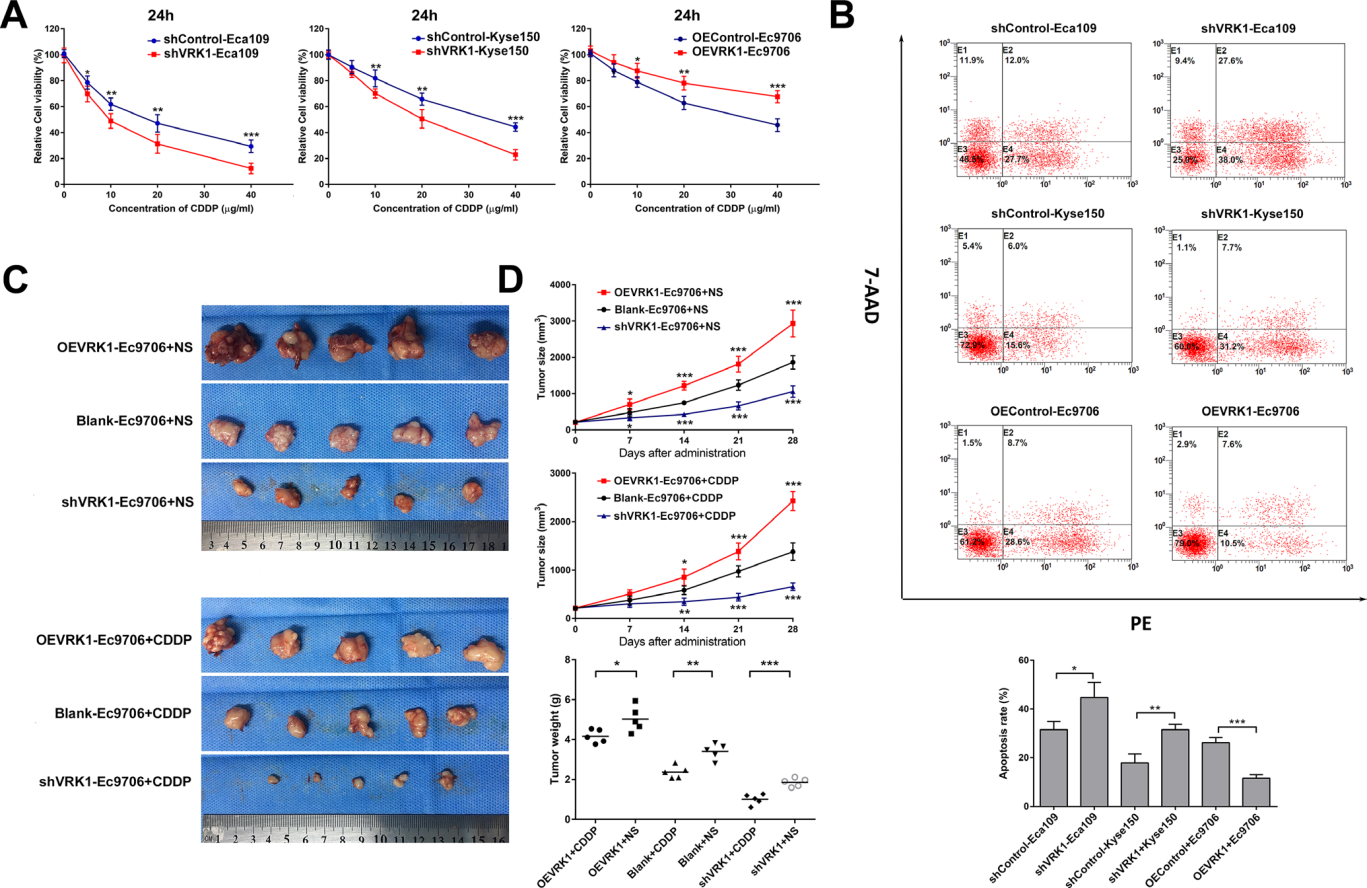
VRK1 promotes CDDP resistance in ESCC both *in vitro* and *in vivo* (**A**) The survival rates of the indicated ESCC cells after treatment with CDDP for 24 h were assessed with a Cell Counting Kit-8 (CCK-8) assay. (**B**) After treatment with 10 μg/ml CDDP for 24 h, apoptosis of the indicated ESCC cells was detected with flow cytometry. (**C**, **D**) Representative photograph (C) of fresh tumor tissues and quantification analysis of tumor sizes (D) in the xenograft model. Overexpression of VRK1 promoted, whereas knockdown of VRK1 inhibited, tumor growth and CDDP resistance *in vivo*. Statistical analyses were performed using one-way ANOVA or Student’s *t*-test. The results are expressed as the mean ± SD of three independent experiments; *P < 0.05, **P < 0.01, and ***P < 0.001.

As indicated by a cell viability assay, reconstitution of VRK1 expression in Ec9706 cells notably elevated cell survival relative to that of the control cells in response to CDDP in a dose- and time-dependent manner. Conversely, knockdown of endogenous VRK1 markedly sensitized Eca109 and Kyse150 cells to CDDP (Figure 3A, Supplementary Figure 2A). Moreover, we subjected ESCC cells that stably expressed either VRK1 or VRK1 shRNA to apoptosis analysis using flow cytometry. Concordantly, overexpression of VRK1 inhibited ESCC cell apoptosis in response to CDDP, whereas silencing VRK1 promoted apoptosis (Figure 3B).

In parallel with the observed VRK1-mediated CDDP resistance *in vitro*, a xenograft model showed that tumors formed by OEVRK1-Ec9706 cells were larger than those formed by Blank-Ec9706 cells in both the normal saline (NS) and CDDP administration groups. In contrast, tumors formed by shVRK1-Ec9706 cells exhibited a smaller size and lower mass than control tumors. Tumors with elevated VRK1 expression presented increased chemoresistance to CDDP, while tumors with down-regulated VRK1 expression exhibited a marked cytotoxic response to CDDP (Figure 3C, 3D and Supplementary Figure 2B). Taken together, these observations indicated that VRK1 counteracted the anti-tumor effects of CDDP.

### c-Jun phosphorylation by VRK1 is essential for VRK1-induced chemoresistance

**Figure 4:**
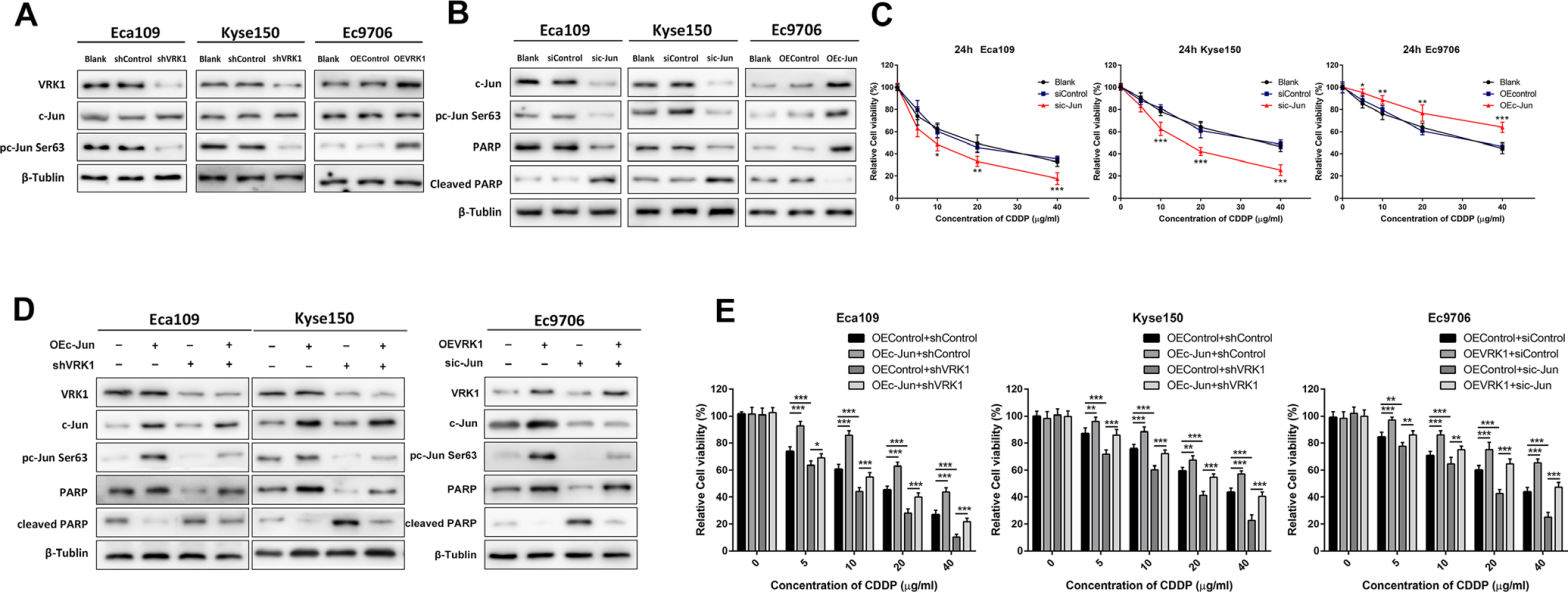
VRK1 induces CDDP resistance by activating and phosphorylating c-Jun in ESCC (**A**) Western blot showing that inhibition of VRK1 in Eca109 and Kyse150 cells suppressed c-Jun phosphorylation, while VRK1 overexpression in Ec9706 cells promoted c-Jun phosphorylation. (**B**) c-Jun knockdown enhanced CDDP-mediated apoptosis, and vice versa. The indicated cells were treated with 10 μg/ml CDDP for 24 h and then subjected to western blotting. Apoptosis was determined by examining the level of cleaved-PARP; β-tubulin was used as an internal control. (**C**) A CCK-8 assay demonstrated that c-Jun promoted CDDP resistance in ESCC cells. (**D**) The increased apoptosis in response to 10 μg/ml CDDP in Eca109 and Kyse150 cells stably expressing VRK1-shRNA was reduced by up-regulation of c-Jun. c-Jun knockdown in Ec9706 cells blocked the anti-apoptotic effect of VRK1 overexpression upon CDDP administration. (**E**) The viability of the indicated cells treated with a series of CDDP concentrations for 24 h was measured with a CCK-8 assay. Overexpression of c-Jun re-sensitized ESCC cells transduced with VRK1-shRNA to CDDP. Inhibition of c-Jun blocked VRK1-mediated resistance to CDDP in ESCC cells. Statistical analyses were performed using one-way ANOVA or Student’s *t*-test. The results are expressed as the mean ± SD of three independent experiments; *P < 0.05, **P < 0.01, and ***P < 0.001.

The role of c-Jun in the progression of various cancers has been elucidated in several studies. Phosphorylated c-Jun becomes transcriptionally active by forming the transcription factor AP-1 via interaction with other transcription factors and therefore inducing resistance to the lethal effects of some cytotoxic drugs [27–29]. A previous study showed that c-Jun could be phosphorylated by VRK1 at Ser63 and Ser73 [24]. To explore whether VRK1 phosphorylates c-Jun in ESCC to mediate CDDP resistance, western blotting was performed to assess the total protein and phosphorylated protein levels in ESCC cell lines. As shown in Figure 4A, the proportion of phosphorylated c-Jun was consistently up-regulated in VRK1-transduced ESCC cells and decreased in VRK1-silenced ESCC cells, suggesting a potential role of c-Jun in VRK1-mediated CDDP resistance.

As predicted, silencing c-Jun in Eca109 and Kyse150 cells sensitized these cells to CDDP, decreased cell proliferation (Figure 4C, Supplementary Figure 3) and promoted cell apoptosis (Figure 4B). Similarly, Ec9706 cells stably overexpressing c-Jun displayed reduced sensitivity to CDDP, increased proliferation (Figure 4C, Supplementary Figure 3) and decreased apoptosis (Figure 4B). Moreover, c-Jun inhibition decreased the ability of Ec9706 cells with constitutive VRK1 overexpression to induce CDDP resistance according to CCK-8 and apoptosis assays. Meanwhile, the increased responsiveness to CDDP was reversed in Eca109 and Kyse150 cells transfected with VRK1-shRNA through c-Jun overexpression (Figure 4D, 4E).

### The VRK1/c-Jun pathway promotes CDDP resistance in ESCC by activating c-MYC

**Figure 5:**
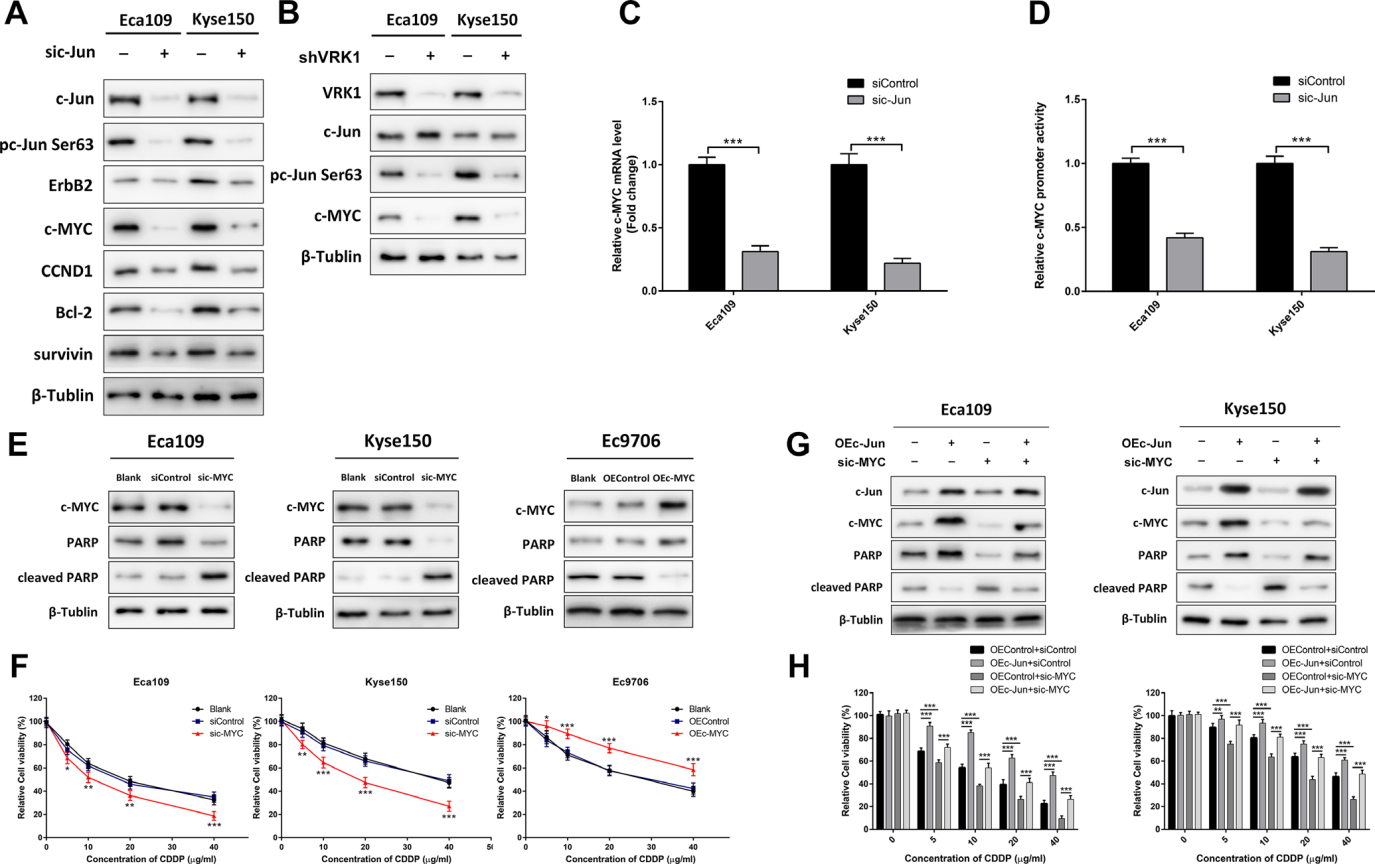
VRK1/c-Jun promotes CDDP resistance via up-regulation of c-MYC in ESCC (**A**) Western blot showing that knockdown of c-Jun reduced c-MYC expression, and inhibition of c-Jun also suppressed the protein levels of ErbB2, CCND1, Bcl-2 and survivin. (**B**) Depletion of VRK1 down-regulated c-MYC expression. (**C, D**) Knockdown of c-Jun decreased the mRNA level of c-MYC determined by RT-PCR (C) and luciferase activity of the c-MYC promoter in a dual luciferase reporter system (D). (**E**) c-MYC knockdown enhanced CDDP-mediated apoptosis, and vice versa. The indicated cells were treated with 10 μg/ml CDDP for 24 h and then subjected to western blotting. Apoptosis was determined by examining the level of cleaved-PARP; β-tubulin was used as an internal control. (**F**) A CCK-8 assay demonstrated that c-MYC promoted CDDP resistance in ESCC cells. (**G**) The reduced apoptosis in response to 10 μg/ml CDDP in Eca109 and Kyse150 cells stably expressing c-Jun was rescued by knockdown of c-MYC. (**H**) The viability of the indicated cells treated with a series of CDDP concentrations was measured with a CCK-8 assay. Inhibition of c-MYC blocked c-Jun-mediated resistance to CDDP in ESCC cells. Statistical analyses were performed using one-way ANOVA or Student’s *t*-test. The results are expressed as the mean ± SD of three independent experiments; *P < 0.05, **P < 0.01, and ***P < 0.001.

We further dissected the molecular mechanism by which phosphorylated c-Jun enhanced the chemoresistance of ESCC cells to CDDP by detecting the protein levels of some CDDP resistance-related genes in Eca109 and Kyse150 cells transfected with c-Jun siRNA. We found by western blotting that down-regulation of c-Jun attenuated the expression of survivin, c-MYC, ErbB2, CCND1 and Bcl-2 (Figure 5A). Among these molecules, the protein levels of c-MYC were the most reduced in Eca109 and Kyse150 cells with c-Jun knockdown. Concurrently, RT-PCR showed that inhibition of c-Jun led to a dramatic reduction in c-MYC at the mRNA level (Figure 5C). Moreover, down-regulation of c-MYC was observed in Eca109 and Kyse150 cells with stable VRK1 knockdown, confirming that VRK1 could regulate c-MYC levels by activating c-Jun (Figure 5B).

Furthermore, a dual luciferase reporter system was used to clarify whether c-Jun could activate c-MYC transcription. As shown in Figure 5D, knockdown of c-Jun inhibited the luciferase activity of the c-MYC promoter in ESCC cells, indicating that c-Jun regulated the expression of c-MYC at the transcriptional level.

To elucidate the potential physiological and pathological role of c-MYC in the VRK1/c-Jun pathway, we examined the effect of c-MYC on CDDP resistance in ESCC cells. The results showed that knockdown of c-MYC significantly sensitized Eca109 and Kyse150 ESCC cells to CDDP (Figure 5E, 5F, and Supplementary Figure 4), while Ec9706 cells with constitutive c-MYC overexpression exhibited significantly superior cell viability (Supplementary Figure 5E and Supplementary Figure 4) and less apoptosis (Figure 5F) after CDDP treatment. In addition, silencing c-MYC strongly blocked c-Jun-enhanced CDDP resistance in Eca109 and Kyse150 cells (Figure 5G, 5H). Collectively, these observations confirmed that VRK1 regulated susceptibility to CDDP in ESCC cells by up-regulating c-MYC through c-Jun phosphorylation and activation.

### Luteolin, a VRK1 inhibitor, can attenuate the malignant phenotype in ESCC

**Figure 6:**
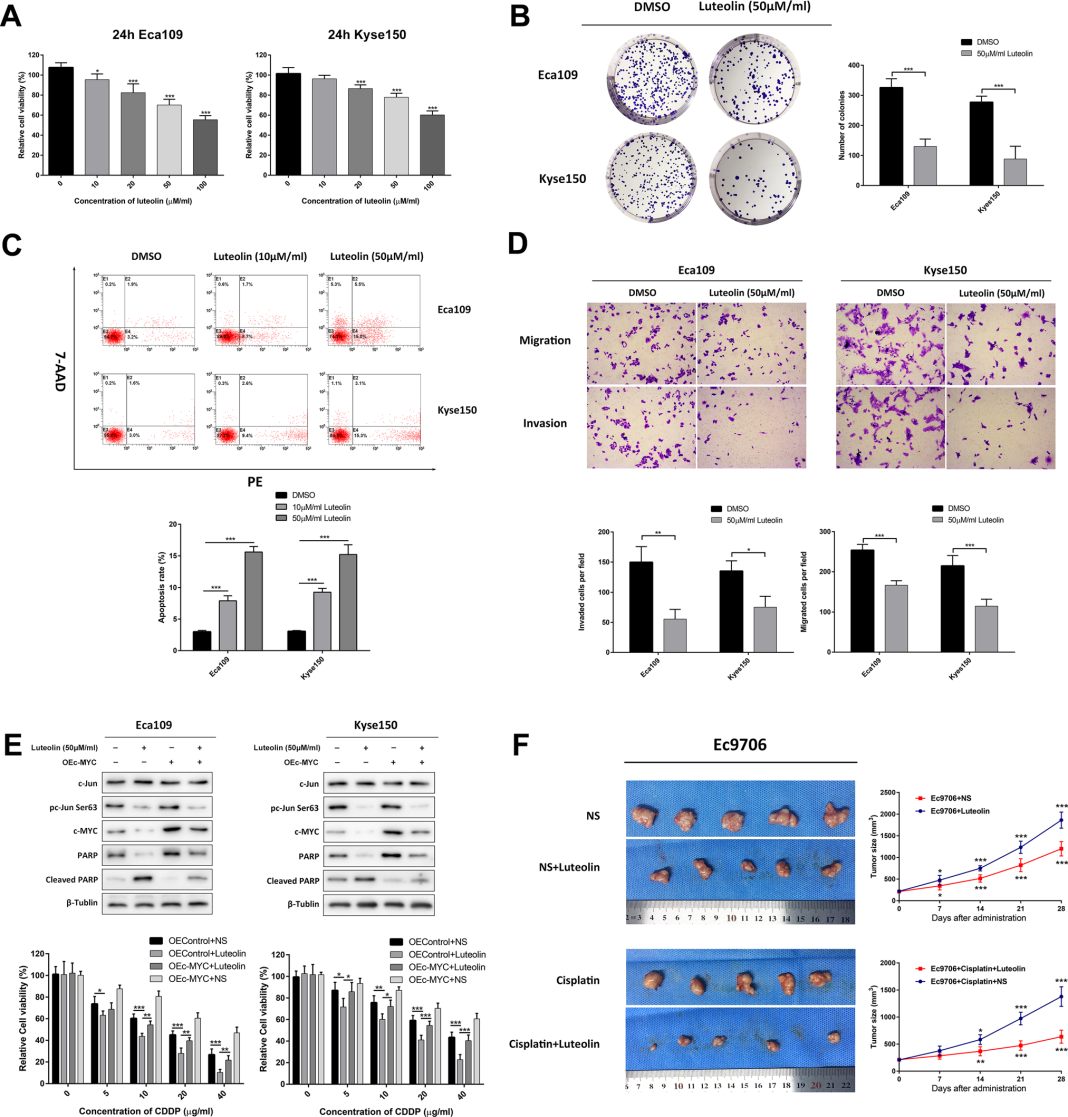
Luteolin inhibits the initiation and progression of ESCC (**A, B**) Luteolin treatment reduced cell viability (A) and colony formation (B) of Eca109 and Kyse150 cells. (**C**) Flow cytometry analysis via PE/7-AAD staining showed that luteolin promoted ESCC cell apoptosis. (**D**) Luteolin suppressed the migration and invasion of Eca109 and Kyse150 cells. (**E**) Luteolin sensitized ESCC cells to CDDP by targeting the VRK1/c-Jun/c-MYC axis, as analyzed by western blot and CCK-8 assay. (**F**) Representative photograph of fresh tumor tissues and quantification analysis of tumor sizes in the xenograft model. Administration of luteolin reduced tumor growth and CDDP resistance *in vivo*. Statistical analyses were performed using one-way ANOVA or Student’s *t*-test. The results are expressed as the mean ± SD of three independent experiments; *P < 0.05, **P < 0.01, and ***P < 0.001.

Luteolin, a naturally occurring flavonoid, has been demonstrated to inhibit VRK1 activity by directly interacting with the catalytic domain of VRK1 [30]. Thus, we examined whether luteolin could simulate the anticancer effect of VRK1 depletion in ESCC. As shown in Figure 6, in response to luteolin, ESCC cell proliferation was significantly reduced (Figure 6A, Supplementary Figure 5) and apoptosis (Figure 6C) was significantly increased in a dose-dependent manner. Similar results were obtained from a colony formation assay (Figure 6B). Additionally, luteolin also suppressed the invasive and migratory ability of Eca109 and Kyse150 cells (Figure 6D).

We next investigated whether luteolin was involved in attenuating CDDP resistance in ESCC. As shown in Figure 6E, luteolin inhibited c-Jun phosphorylation and c-MYC expression. Moreover, as analyzed by western blotting and CCK-8 assay, luteolin enhanced the cytotoxic effect of CDDP on ESCC cells, while overexpression of c-MYC blocked this phenotype. All these results confirmed that administration of luteolin attenuated CDDP resistance via the VRK1/c-Jun/c-MYC axis. Furthermore, to confirm the anticancer effect of luteolin *in vivo*, luteolin was intraperitoneally injected twice a week into nude mice inoculated with Blank-Ec9706 cells. Significant differences in tumor size were observed between the luteolin-treated group and the NS-treated group. Additionally, luteolin administration rendered tumors more sensitive to CDDP than NS administration (Figure 6F).

### VRK1 expression is correlated with c-MYC expression in ESCC

**Figure 7:**
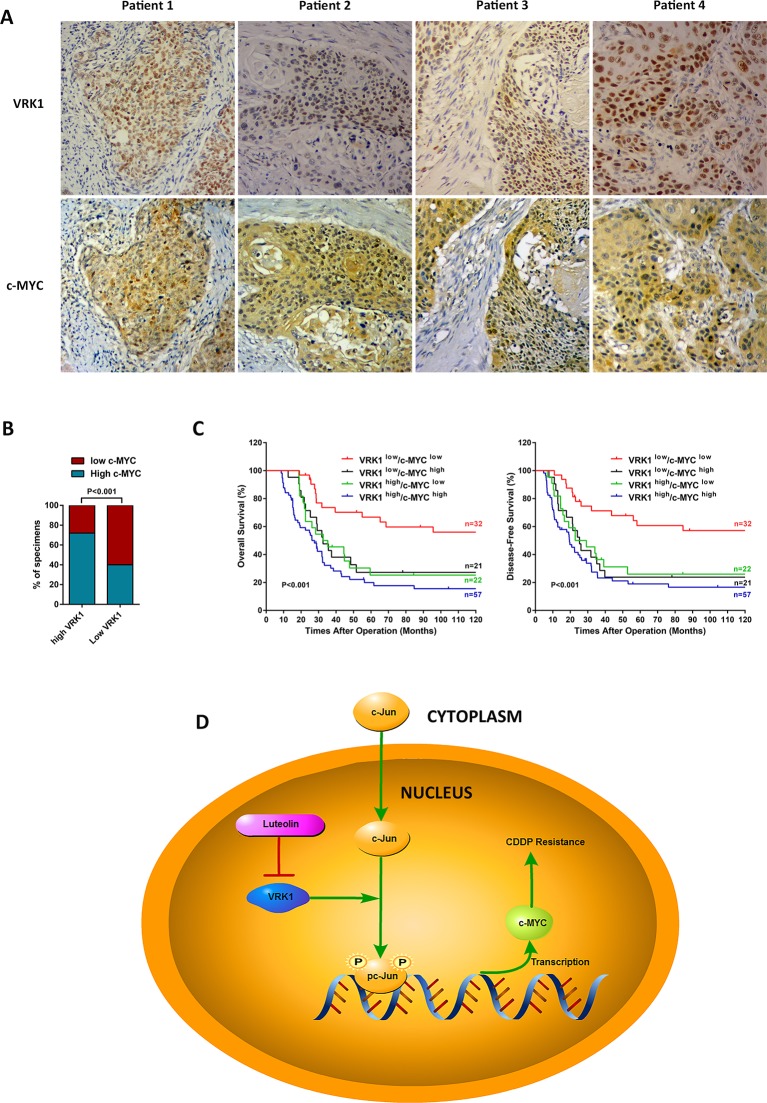
c-MYC is correlated with VRK1 expression and schematic of VRK1/c-Jun/c-MYC pathway in ESCC (**A, B**) Representative immunohistochemistry images (A) and statistical analysis (B) of the relationship between VRK1 levels and c-MYC levels in 132 paraffin-embedded ESCC specimens (*P* < 0.001, Fisher’s exact test). (**C**) A positive correlation between VRK1 and c-MYC expression is a potential prognostic factor in ESCC. High VRK1 and high c-MYC expression, when taken together, were significantly associated with poor OS (*P* < 0.001) and DFS (*P* < 0.001) in comparison with patients with low VRK1 and low c-MYC expression (**D**) Diagram illustrating the VRK1/c-Jun/c-MYC pathway mediating CDDP resistance in ESCC. After translocating from the cytoplasm into the nucleus, nonphosphorylated c-Jun was phosphorylated and activated by VRK1. Activated c-Jun forms homodimers or heterodimers with other transcriptional factors and activates the transcription of c-MYC, leading to CDDP resistance in ESCC. *P < 0.05, **P < 0.01, and ***P < 0.001.

We further validated the interplay between VRK1 and c-MYC in ESCC by performing an IHC analysis. As shown in Figure 7A and 7B, VRK1 expression was strongly correlated with c-MYC expression in 132 ESCC specimens (*P* < 0.001). Moreover, we analyzed the OS and DFS of these 132 patients, who were categorized into four groups according to the expression level of VRK1/c-MYC. Kaplan-Meier analysis revealed that patients in the VRK1^low^/c-MYC ^low^ group exhibited significantly longer OS and DFS in comparison with the other three groups, while patients in the VRK1^high^/c-MYC ^high^ group carried the worst prognosis (Figure 7C). These results further supported the view that VRK1 enhanced the expression of c-MYC, which in turned led to a poorer prognosis in ESCC patients.

## DISCUSSION

VRK1, which was discovered because of its sequence similarity with vaccinia virus B1R kinase, was initially regarded as a regulator of cell division and cell cycle [9, 31]. Recent studies have uncovered the role of VRK1 in cell proliferation and cancer. High VRK1 levels were detected in normal tissues with high proliferation such as the thymus and testis as well as in tumor tissues [31, 32]. However, the exact role of VRK1 in ESCC tumorigenesis or development still requires clarification. In our work, VRK1 was overexpressed in ESCC tissues compared with that in adjacent non-tumor tissues. Moreover, there was a significant correlation between VRK1 expression levels and T stage, lymphatic metastasis and poor outcomes of ESCC patients. The multivariate Cox regression analysis showed that VRK1 was an independent prognostic factor. Furthermore, by either up-regulating or down-regulating VRK1 in ESCC cells, we characterized the cellular aspect of VRK1 function and showed that VRK1 not only promoted cell proliferation and survival but also induced cell invasion and migration in ESCC. Consistent with this, in a xenograft mouse model, overexpression of VRK1 enhanced ESCC tumorigenicity, whereas knockdown of VRK1 reduced this activity. These results suggested that VRK1 potentiated the malignant phenotype of ESCC cells.

Although CDDP alone or in combination with other cytotoxic drugs is recommended as a first-line therapy for patients with advanced ESCC, the application of CDDP is limited owing to chemoresistance [33, 34]. Thus, identifying the exact mechanism of CDDP resistance and developing a treatment to circumvent CDDP resistance remain critical goals for anticancer therapy. Because VRK1 promoted ESCC cell proliferation and survival, we hypothesized that VRK1 plays a crucial role in CDDP resistance in ESCC. In support of our hypothesis, VRK1 inhibition sensitized ESCC cells to CDDP both *in vitro* and *in vivo*; conversely, reconstituting VRK1 made cells refractory to CDDP *in vitro* and *in vivo*. Our findings are the first to describe VRK1 as a promoter of CDDP resistance in ESCC.

Apoptosis triggered by DNA damage is a key step that leads to cell death, while c-Jun, a component of the AP-1 transcription complex, can counteract apoptosis in response to CDDP treatment by activating the transcription of several anti-apoptotic molecules [35, 36]. A previous study found that VRK1 phosphorylates c-Jun at Ser63 and Ser73 and interacts with the N-terminal kinase of c-Jun (JNK); additionally, elevated levels of VRK1 resulted in increased c-Jun phosphorylation at the same sites that JNK phosphorylates, but the phosphorylation increase was independent of JNK activity [24]. In our study, we confirmed that VRK1 could phosphorylate c-Jun in ESCC cells and that this phenomenon plays a critical role in VRK1-mediated CDDP resistance. Thus, we reveal a novel pathway that regulates c-Jun in ESCC independent of JNK. Moreover, many studies support the notion that c-Jun is anti-apoptotic and that this transcription factor could be a promising target to enhance the cytotoxic effect of CDDP [37, 38]. Strangely, JNK, the primary activator of c-Jun, generally seems to be pro-apoptotic in response to CDDP [39–41]. This juxtaposition might be explained by the notion that the anti-apoptotic effect of c-Jun is mediated by its upstream activator VRK1 but not JNK, as the apoptotic role of JNK is carried out via interactions with substrates other than c-Jun. Since VRK1 is mainly localized in the nucleus, the activated VRK1/c-Jun pathway may influence some events that occur in the nucleus (e.g., DNA damage), while the JNK/c-Jun pathway responds to extracellular stresses. c-Jun is phosphorylated by VRK1 and becomes transcriptionally active, and this action may promote the expression of some vital oncogenes that mediate CDDP resistance.

The pro-oncogene c-MYC is one of the genes most frequently involved in cancer pathogenesis. In our experiments, we confirmed that c-MYC was regulated by c-Jun at the transcriptional level and that inhibition of c-Jun led to a dramatic reduction in c-MYC at the mRNA and protein level. Knockdown of c-MYC enhanced the cytotoxic effect of CDDP on ESCC cells, and up-regulation of c-MYC mitigated this effect. c-MYC can strongly block c-Jun-mediated CDDP resistance in ESCC. Despite several studies that have shown that VRK1 contributes to drug resistance by attenuating double-strand breaks (DSBs) through direct regulation of γ-H2AX, NBS1 and 53BP1 foci formation in the induction of the DDR [7, 14, 15], the formation of DNA-DNA inter- and intra-strand adducts, followed by DNA distortion, constitutes the primary mechanism of CDDP-induced DNA lesions rather than DSBs [42, 43]. Thus, we propose the VRK1/c-Jun/c-MYC axis as a novel pathway that contributes to CDDP resistance in ESCC, and the components of the DNA repair system, including γ-H2AX, NBS1 and 53BP1, which are regulated directly by VRK1, may account for other types of DNA damage, such as ionizing radiation or other chemotherapeutic agents.

The development of effective and selective treatments for ESCC is complicated and challenging because of the absence of a defined target. Here, we used luteolin, a VRK1inhibitor, to mimic the inhibitory effect of VRK1 knockdown in both *in vitro* and *in vivo* assays. Our studies revealed that luteolin suppressed ESCC cell proliferation, survival, invasion, and migration as well as CDDP resistance.

Taken together, we suggest that some undefined stress in the nucleus causes the levels of nuclear c-Jun to increase and that c-Jun is phosphorylated by aberrant VRK1 overexpression to become transcriptionally active. Phosphorylated c-Jun then initiates c-MYC transcription and eventually leads to CDDP resistance in ESCC cells (Figure 7D).

Our study also has some limitations. First, the study was based on patients from one center, which makes it difficult to avoid bias. Second, more appropriate studies should be performed to further examine the anticancer effect of luteolin and explore whether luteolin could be applied clinically to treat ESCC. Finally, in our previous study, c-Jun knockdown barely rescued the VRK1-enhanced invasive phenotype of ESCC cells that constitutively overexpressed VRK1, which indicated that signaling pathways other than VRK1/c-Jun/c-MYC might mediate the pro-invasive effect of VRK1 in ESCC. Thus, more effort should be exerted to uncover the mechanism underlying the VRK1-induced invasive phenotype of ESCC.

In summary, our present work provides the first evidence that VRK1 plays an essential role in the initiation and progression of ESCC as well as the induction of CDDP resistance via c-Jun-mediated c-MYC activation. Our findings indicate that VRK1 can serve as a potential therapeutic target for treating ESCC.

## MATERIALS AND METHODS

### Ethical statement

This study was approved by the ethics committee of Shandong Provincial Hospital Affiliated to Shandong University. Written informed consent was obtained from each patient prior to enrollment in this study. All animal experiments were conducted in accordance with ethical standards, the Declaration of Helsinki and national and international guidelines and were approved by the Animal Care and Use Committee.

### Clinical samples and cell lines

A total of 80 surgical specimens were collected from patients subjected to esophagectomy in Shandong Provincial Hospital Affiliated to Shandong University between 2013 and 2016. Patients who underwent incomplete resection, received preoperative chemotherapy or radiotherapy, experienced severe perioperative complications or were lost to follow up and those who died of non-cancer-related causes were excluded. Thus, fresh surgical tumor samples and coupled ANCTs from 41 eligible ESCC patients were collected for this study according to the inclusion criteria stated below. The anatomical location of tumors included upper-thoracic (6/41), mid-thoracic (25/41), and lower-thoracic (10/41) regions. The ANCTs obtained were more than 5 cm away from the tumor margin. None of the ANCTs exhibited tumor infiltration, deterioration or necrosis based on both macroscopic and microscopic examination. All samples were collected at the time of surgical resection and were immediately frozen in liquid nitrogen until further use.

For immunohistochemistry, a total of 132 paraffin-embedded ESCC specimens, which had been collected from 72 patients who underwent biopsy and 60 patients who underwent curative esophagectomy at Shandong Provincial Hospital Affiliated to Shandong University from 2004 to 2005, were employed in the analysis. Patients were followed up every 3 months during the first postoperative year and every 6 to 12 months thereafter for survival or recurrence inquiry until death or the end of this study.

Patient eligibility criteria were as follows: (1) ESCC diagnosis was confirmed by two pathologists; (2) each patient received curative esophagectomy with lymph node dissection, and no residual cancer cells were observed outside the upper or lower cutting edge or outside the lateral margins (3) no preoperative chemotherapy or radiotherapy; (4) no severe perioperative complications; (5) no synchronous or metachronous cancers besides ESCC; (6) each patient was followed up regularly for 3 months after the operation. All patients were staged pathologically after the operation based on the International Union Against Cancer (UICC)/TNM Classification of Malignant Tumors [44].

The human esophageal squamous carcinoma cell lines Eca109, Kyse150, Kyse410, Kyse450, TE-1, and EC9706 and the normal esophageal epithelial cell line Het-1a, and HECC were purchased from Chinese Academy of Science cell bank (Shanghai, China). The cell lines were grown in RPMI 1640 medium (HyClone, USA) supplemented with 10% fetal bovine serum (Gibco, USA), 100 U/ml penicillin and 100 µg/ml streptomycin in a humidified 37°C incubator with 5% CO_2_ and 95% O_2_.

### Real-time quantitative PCR

Total RNA was extracted from cell lines and tissue samples with RNAiso (Takara Bio, Japan) and was stored in RNAsaver (Solarbio, China) at −20°C until use. RNA concentration was measured with a NanoDrop 2000 spectrophotometer (Thermo Fisher, USA). Then, 1 µg of RNA was reverse-transcribed into complementary DNA on an S1000 Thermal Cycler (Bio-Rad, USA). The mRNA expression level was determined using SYBR-Green technology (Takara Bio, Japan) and analyzed on a Roche 480 System (Roche, Switzerland) according to the manufacturer’s instructions [45]. β-Actin was used as an internal standard to minimize errors. Normalized results are expressed using the 2^-∆∆CT^ method. The primers for RT-PCR are shown in Supplementary Table 1. All experiments were performed in triplicate and repeated three times.

### Western blot analysis

Western blot analysis was performed as previously described [46]. Cells were collected and lysed in RIPA buffer (Solarbio, China) plus phenylmethysulfonyl fluoride (PMSF) on ice for 30 min, followed by centrifugation at 12000 g for 20 min. The protein concentration in the extract was measured with a BCA Protein Assay Kit (Pierce, USA). After boiling for 10 min to denature the protein, the samples were diluted in 4× loading buffer (Beyotime, China) and loaded onto SDS-PAGE (10%) gels for electrophoresis and transferred to PVDF membranes (Millipore, USA). BenchMark Pre-Stained Protein Standard (Invitrogen, USA) was used as the size marker. The membranes were blocked with TBST containing 5% non-fat milk for 1 hour, followed by immunoblotting with primary antibodies at 4°C overnight. After incubation with HRP-conjugated secondary antibody (Zhongshan Goldenbridge Biotechnology Company, China) for 1 h, the protein bands were evaluated using enhanced chemiluminescence (Millipore, USA) and detected with a LAS-4000 MINI System (GE, USA). β-Actin or β-tubulin was used as an endogenous control. All the experiments were repeated three times. Antibodies for VRK1 (1:100000), c-Jun (1:1000), phospho-c-Jun (Ser63) (1:5000), c-MYC (1:10000), β-actin (1:1000) and β-tubulin (1:1000) were purchased from Abcam.

### Immunohistochemistry

Immunohistochemistry was performed as previously described [46]. In brief, paraffin-embedded slides were deparaffinized in xylene and rehydrated in a graded alcohol series. Antigen retrieval via microwave treatment in citrate buffer (pH = 6.0) for 10 min was carried out, followed by incubation with 3% H_2_O_2_ for 20 min at room temperature to block endogenous peroxidase. The slides were incubated with the primary antibody for VRK1 (Abcam, 1:150) or c-MYC (Abcam, 1:250) at 4°C overnight. On the next day, the slides were treated with the secondary biotinylated antibody (Zhongshan Goldenbridge Biotechnology Company, China) and subsequently the avidin-biotin complex reagent. The nuclei were stained with hematoxylin.

Three representative staining fields in each section were evaluated by two independent pathologists who were unaware of the patient information; a final consensus score was determined by the two pathologists to avoid any discrepancies. In general, the SI of VRK1 or c-MYC was determined by combining the staining intensity score (1, negative; 2, weak staining; 3, moderate staining; 4, strong staining) and the proportion of positively stained tumor cells (0, no positive cells; 1, < 10%; 2, 10%–35%; 3, 35%–75%; 4, > 75%). Samples with SI ≥ 8 were considered to exhibit high expression, while samples with SI < 8 were deemed to be low expression samples [47].

### Plasmid construction, transfection and small interfering RNA

To explore the biological function of VRK1, a CMV-VRK1-EGFP-SV40-Neomycin plasmid was constructed to induce VRK1 overexpression. To silence endogenous VRK1, GV248-small hairpin VRK1 (shVRK1) and GV248-small hairpin (shcontrol) RNAs were generated by inserting the corresponding oligonucleotides into a GV248-puro lentiviral vector (Genechem, Shanghai, China). The siRNA sequences targeting c-Jun and c-MYC were synthesized by Ribobio (Shanghai, China). Oligonucleotides sequences for silencing target genes are shown in Supplementary Table 2. The c-Jun and c-MYC expression plasmids CMV-c-Jun-EGFP-SV40-Neomycin and CMV-c-MYC-EGFP-SV-40-Neomycin, respectively, were acquired from Genechem (Shanghai, China).

Transfection of siRNA or the overexpression plasmid was performed in 60%–70% confluent cells using Lipofectamine 2000 reagent (Invitrogen, USA) according to the manufacturer’s protocol. Stable cell lines overexpressing VRK1, c-Jun or c-MYC were selected with 200 μg/ml G418 for two weeks. For lentivirus-mediated transduction, ESCC cells were infected with lentivirus encoding Negative control (NC) shRNA or VRK1 shRNA at an MOI of 50 using Polybrene (Genechem, Shanghai, China). Infected cells were selected for two weeks with puromycin at a concentration of 10 μg/ml.

### Luciferase assay

The luciferase assay was performed as reported previously [47]. In brief, 60%–70% confluent cells were transfected with siControl or sic-Jun in triplicate in 48-well plates. After 24 h of transfection, cells were co-transfected with pGL4.10 firefly luciferase plasmid (Promega, USA) containing the c-MYC promoter and pRL-SV40 Renilla luciferase plasmid (Promega, USA) using Lipofectamine 2000 reagent (Invitrogen, USA). The firefly luciferase and Renilla luciferase activities were detected 36 h after transfection using a dual luciferase reporter assay system (Promega, USA) according to the manufacturer’s protocol. The pRL-SV40 Renilla luciferase plasmid was used as an internal control. The effect of c-Jun-mediated transcriptional activity was determined by the ratio of firefly luciferase activity to Renilla luciferase activity. Each assay was performed in triplicate and repeated three times.

### Apoptosis assay

Apoptosis was detected with a flow cytometry assay [48]. Briefly, 5 × 10^5^ cells in the exponential growth period were harvested with trypsin and then washed with PBS three times. The suspended cells were stained with Annexin V-PE and 7-AAD (BD Biosciences, USA) according to the manufacturer’s instructions. The dual PE/7-AAD staining was analyzed with a FACScan flow cytometer (Beckman coulter, USA). The cells with Annexin V-PE-positive and 7AAD-negative were considered as apoptotic cells. Each assay was performed in triplicate three times.

### Cell viability assay

Cell viability was measured using Cell Counting Kit-8 (Dojindo Laboratory, Japan) according to the instructions [49]. Briefly, infected cells were seeded into 96-well plates at a concentration of 5000 cells per well with 100 μl of RPMI 1640 medium supplemented with 10% FBS and with different concentrations of CDDP or luteolin. Cells were cultured for different time lengths. After the culture medium was removed, 10 μl of CCK-8 reagent with 90 μl of fresh 10% FBS medium was added into each well, and the cells were incubated at 37°C for 2 h. The absorbance of each well was detected at 450 nm with a microplate reader (Molecular Devices, USA). The experiment was repeated with each group three times.

### Migration and invasion assays

Transwell insert chambers with 8-μm pores (Corning, USA) were used to carry out migration and invasion assays [50]. For the migration assay, 5 × 10^4^ ESCC cells suspended in RPMI 1640 supplemented with 1% FBS and 100 ng/ml Nocodazole (Sigma-Aldrich, USA) were added to the upper chamber, while for the invasion assay, 5 × 10^4^ cells suspended in RPMI 1640 supplemented with 1% FBS and 100 ng/ml Nocodazole were seeded into the membrane of the insert coated with 300 ng/ml Matrigel (BD Biosciences, USA). Medium containing 10% FBS in the lower chamber served as a chemoattractant. After incubation for 24 h in a humidified environment at 37°C, cells remaining on the top surface of the insert were removed by wiping with a cotton swab. Cells on the lower surface of the filter were stained with 0.2% crystal violet. To control the variation in cell proliferation after VRK1 alteration, Nocodazole was added to arrest cell cycle in M-phase. The cells on the lower surface of the membrane were counted in three independent fields at ×200 magnification. Each assay was repeated three times.

### Colony formation assay

Cells were seeded into 6-well plates with 800 cells per well. Culture medium was replaced regularly, and the cells were allowed to grow for 4 weeks after seeding. Then, the cells were fixed with methanol and stained with 0.2% crystal violet. Colonies containing more than 50 cells were considered valid colonies and were counted [51]. The experiments were repeated three times.

### Wound-healing assay

The wound-healing assay was performed as previously reported [52]. Cells were seeded into 6-well plates at a concentration of 5 × 10^5^ cells per well. Each well was scratched with a pipette tip when the incubated cells had grown to 75% confluence. Cells were photographed at 0 h, 8 h and 24 h. The wound gaps were digital quantified using Image Pro Plus software (Media Cybernetics, USA). The migration index represents migration speed in relative to control group. Each assay was repeated three times.

### Xenograft model and *in vivo* chemosensitivity to CDDP

Forty female 3- to 4-week-old BALB/c nude mice were purchased from SLAC Laboratory Animal, Chinese Academy of Sciences (Shanghai, China). All the experiments were conducted under the specific pathogen-free conditions at the Laboratory Animal Center of Shandong Provincial Hospital. Nude mice were randomly divided into the eight groups (*n* = 5 for each group) shown in Table 1. The indicated Ec9706 cells (3 × 10^6^) suspended in 0.1 mL of NS were subcutaneously implanted into the right axilla of nude mice anesthetized with sodium pentobarbital. The two largest perpendicular diameters of the tumors were measured with calipers every week. As soon as the tumor volumes reached 200 mm^3^, the subjects of the corresponding group were further administered 2 mg/kg CDDP, 5 mg/kg luteolin or NS twice a week, as indicated in Table 3. Four weeks after administration, the mice were sacrificed and the tumors were excised and measured.

**Table 3:** Detailed treatments of each group in the xenograft model

Group (5 nude mice)	Cells inoculated	CDDP (2 mg/kg)	NS	Luteolin (5 mg/kg)
OEVRK1-Ec9706+CDDP	OEVRK1 -Ec9706	+	-	-
OEVRK1-Ec9706+NS		-	+	-
shVRK1-Ec9706+CDDP	shVRK1-Ec9706	+	-	-
shVRK1-Ec9706+NS		-	+	-
Blank-Ec9706+CDDP	Blank-Ec9706	+	-	-
Blank-Ec9706+NS		-	+	-
Blank-Ec9706+CDDP+Luteolin		+	-	+
Blank-Ec9706+NS+Luteolin		-	+	+

Abbreviations: VRK1, vaccinia related kinase 1; CDDP, cisplatin; NS, normal saline; OEVRK1-Ec9706, Ec9706 stably overexpressing VRK1; shVRK1-Ec9706, Ec9706 cells stably expressing VRK1-shRNA.

### Statistical analysis

All the statistical analyses were performed with SPSS19.0 for Windows (IBM, USA) in a two-sided manner. For comparisons, Student’s *t*-test or one-way ANOVA was used to assess differences among groups. Associations between categorical variables were analyzed with Fisher’s exact test. Survival curves were estimated using the Kaplan-Meier method and log-rank test. The prognostic significance factors were tested in a Cox regression model using univariate and multivariate analyses. All experiments were repeated in triplicate. All data are presented as the means ± SD, and *P* < 0.05 was considered statistically significant (*P < 0.05, **P < 0.01, ***P < 0.001).

## SUPPLEMENTARY MATERIALS FIGURE AND TABLES



## Abbreviations

ESCC: esophageal squamous cell carcinoma
VRK1: vaccinia related kinase 1
CDDP: cisplatin
NS: normal saline
DSB: double-strand breaks
OS: overall survival
DFS: disease-free survival
