# Supplementary material for: VRK1 promotes cisplatin resistance by up-regulating c-MYC via c-Jun activation and serves as a therapeutic target in esophageal squamous cell carcinoma
Source: Oncotarget. 2017 Aug 7;8(39):65642–58. doi: 10.18632/oncotarget.20020 (PMC5630360; doi:10.18632/oncotarget.20020)
Supplement: Supplementary file 1 [file oncotarget-08-65642-s001.pdf]

VRK1 promotes cisplatin resistance by up-regulating c-MYC via c-Jun activation and serves as a therapeutic target in esophageal squamous cell carcinoma

SUPPLEMENTARY MATERIALS

Supplementary Table 1: Primers for quantitative real-time PCR

Primers	Sequences (From 5′ to 3′)
β-Actin-plus	TGCATTGCCGTCAACTTGTG
β-Actin-minus	GGTTCGGCACACCAAAATCC
VRK1-plus	AATTGGGGCAACACGAAAGC
VRK1-minus	CATACACTCCGGGATCTGGC
c-Jun-plus	TAGGTGCATGCGACGGTATC
c-Jun-minus	CGACGGTTGGGACTCTGAAA
c-MYC-plus	TTTGCTGCTGTCATCATGCG
c-MYC-minus	TAACGTCACACGAACCGACA

Supplementary Table 2: Oligonucleotides for silencing the expression of target gene

Target Gene	Sequences (from 5′ to 3′)	Type
VRK1	GAAGTAAGGATGATGGCAAAT	shRNA
c-Jun	TTACTGTAGCCATAAGGTCCG	siRNA
c-MYC	CCTGAGACAGATCAGCAACAA	siRNA

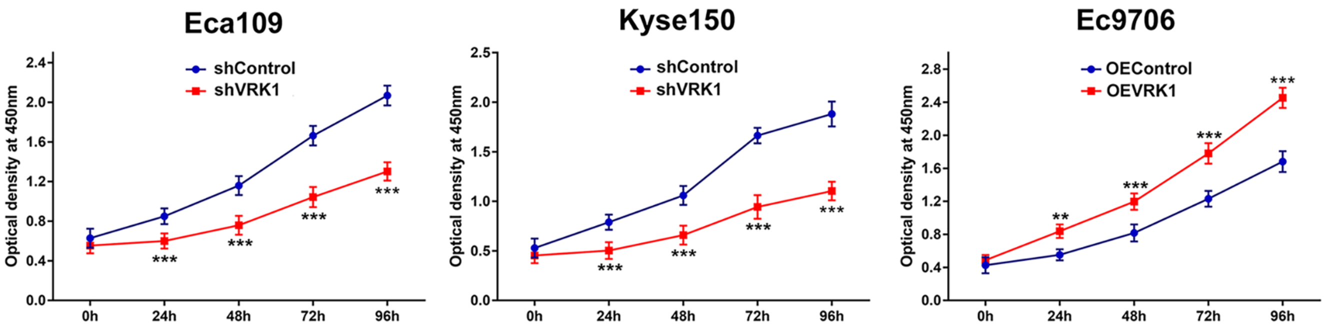

Supplementary Figure 1: VRK1 promotes ESCC cells proliferation. CCK-8 assay showed knockdown of VRK1 suppressed, whereas overexpression of VRK1 promoted, the proliferation and viability of ESCC cells in a time-dependent manner. Statistical analyses were performed using Student's *t*-test. The results are expressed as the mean ± SD of three independent experiments; *$P < 0.05$, **$P < 0.01$, and ***$P < 0.001$.

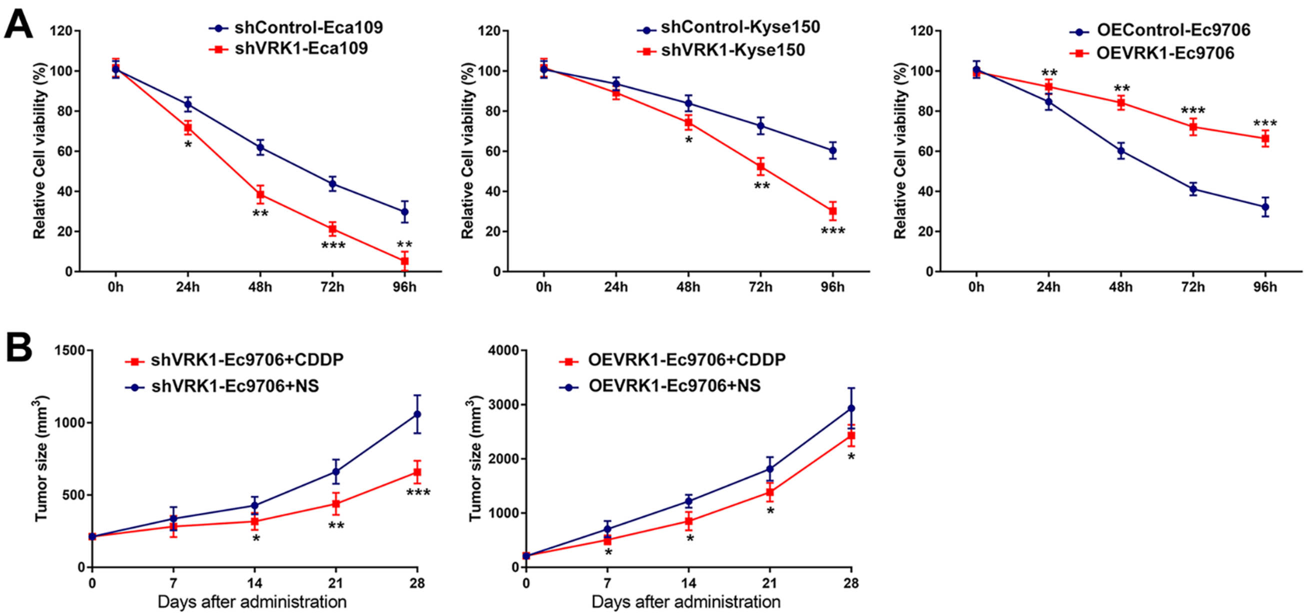

Supplementary Figure 2: VRK1 enhances CDDP resistance both *in vitro* and *in vivo*. (**A**) The viability of indicated cells was analyzed by CCK-8 assay in response to 5μg/ml CDDP in time series. (**B**) The illustration of tumors size in VRK1 knockdown (left) and VRK1 overexpression (right) compare to the same conditions treated with 10μg/ml CDDP. Statistical analyses were performed using Student's *t*-test. The results are expressed as the mean ± SD of three independent experiments; *$P < 0.05$, **$P < 0.01$, and ***$P < 0.001$.

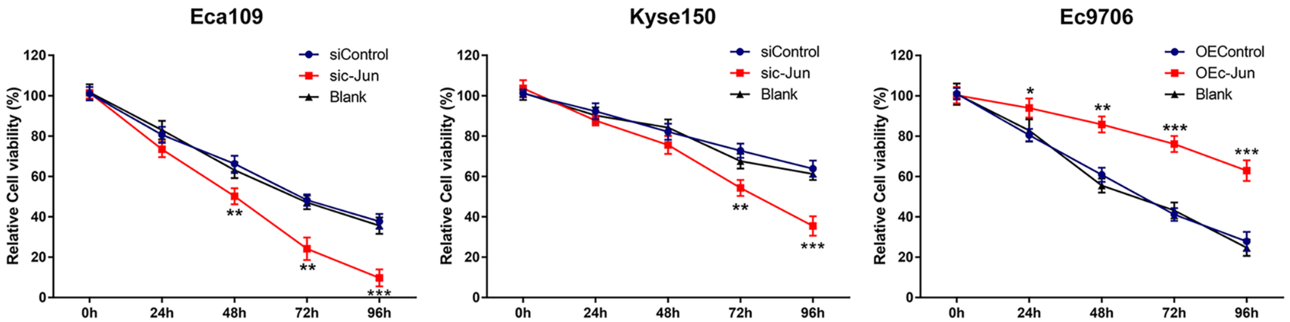

Supplementary Figure 3: c-Jun enhances CDDP resistance in ESCC cells. The viability of indicated cells was analyzed by CCK-8 assay in response to 5 μg/ml CDDP in time series. Statistical analyses were performed using Student's *t*-test. The results are expressed as the mean ± SD of three independent experiments; $^{*}P < 0.05$, $^{**}P < 0.01$, and $^{***}P < 0.001$.

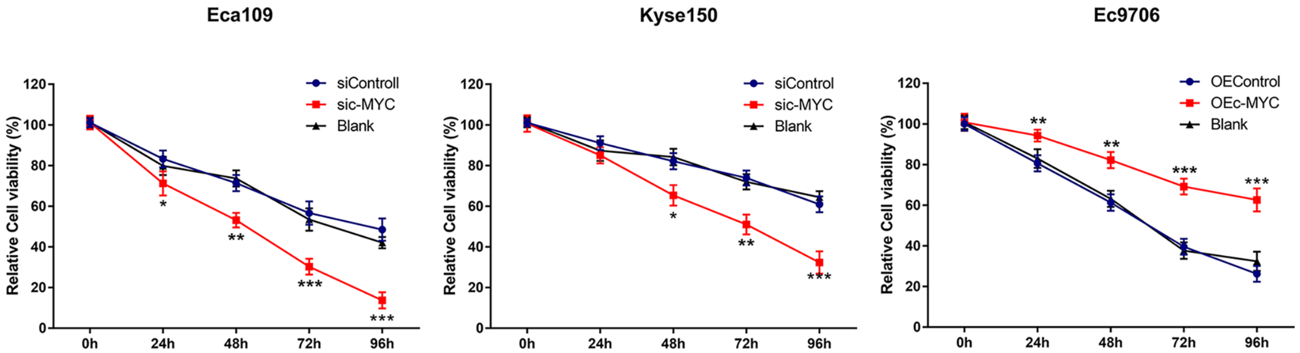

Supplementary Figure 4: c-MYC enhances CDDP resistance in ESCC cells. The viability of indicated cells was analyzed by CCK-8 assay in response to 5μg/ml CDDP in time series. Statistical analyses were performed using Student's *t*-test. The results are expressed as the mean ± SD of three independent experiments; $^{*}P < 0.05$, $^{**}P < 0.01$, and $^{***}P < 0.001$.

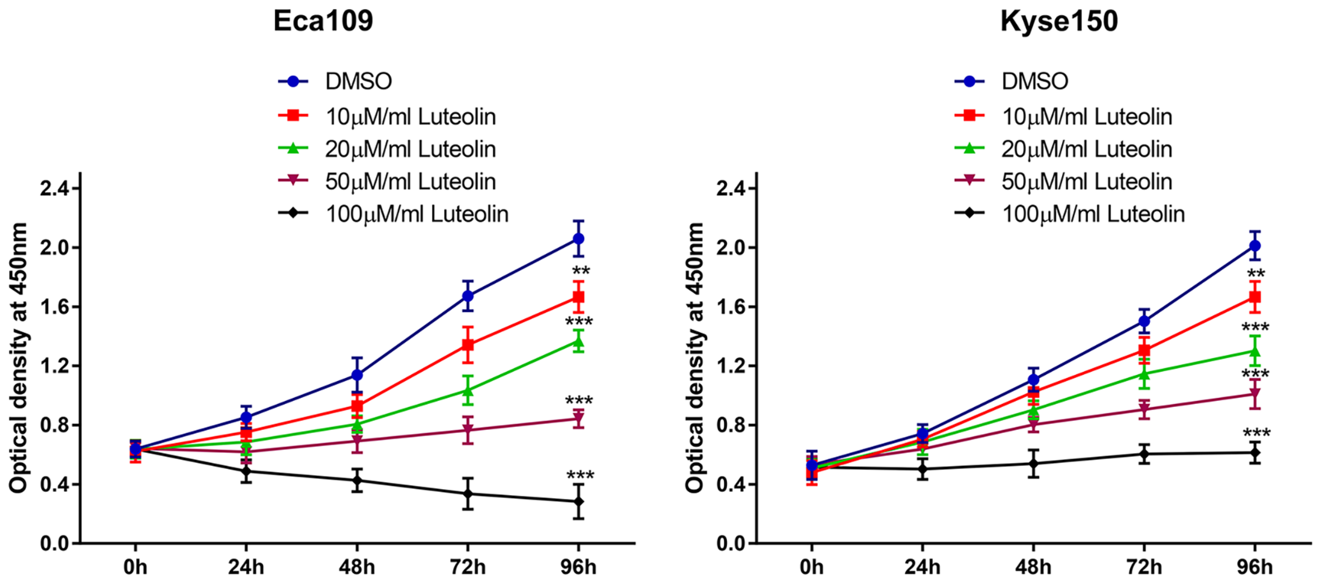

Supplementary Figure 5: Luteolin inhibits proliferation of ESCC cells. Luteolin treatment significantly reduced cell viability of Eca109 and Kyse150 cells in dose- and time- dependent manner, compared with the control group treated with DMSO. Statistical analyses were performed using One-way ANOVA. The results are expressed as the mean ± SD of three independent experiments; $^{*}P < 0.05$, $^{**}P < 0.01$, and $^{***}P < 0.001$.
